# Spleen Perfusion as an Index of Gender Impact on Sympathetic Nervous System Response to Exercise

**DOI:** 10.3389/fphys.2021.780713

**Published:** 2021-12-15

**Authors:** Francesco Lanfranchi, Francesca D'Amico, Stefano Raffa, Michele Pennone, Maria Isabella Donegani, Alberto Miceli, Silvia Chiola, Sara Maggio, Carlo Delucchi, Vanessa Cossu, Silvia Morbelli, Matteo Bauckneht, Gianmario Sambuceti, Cecilia Marini

**Affiliations:** ^1^Nuclear Medicine Unit, Department of Health Sciences, University of Genoa, Genoa, Italy; ^2^IRCCS Ospedale Policlinico San Martino, Genoa, Italy; ^3^CNR Institute of Molecular Bioimaging and Physiology (IBFM), Milan, Italy

**Keywords:** myocardial perfusion imaging, ^99m^Tc-sestamibi, spleen, ischemia, sympathetic nervous system, gender, exercise, stress test

## Abstract

**Objective:** Sympathetic nervous system (SNS) reaction to exercise is gender dependent. Nevertheless, clinically applicable methods to identify this difference are still missing. An organ largely sensitive to SNS is the spleen whose response to exercise can be easily evaluated, being included in the field of view of myocardial perfusion imaging (MPI). Here, we aimed to verify whether gender interferes with the spleen perfusion and its response to exercise.

**Methods:** For this purpose, we evaluated 286 original scans of consecutive patients submitted to MPI in the course of 2019. Our standard procedure implies a single-day stress-rest sequence with a gap of ≥2 h between the administrations of 180 and 500 MBq of ^99m^Tc-Sestamibi, respectively. Imaging is performed 30 min after radiotracer administration, with scan duration set at 25 and 35 s per view, respectively. Non-gated scans were reconstructed with the filtered back-projection method. A volume of interest was drawn on the spleen and heart to estimate the dose-normalized average counting rate that was expressed in normalized counts per seconds (NCPS).

**Results:** In all subjects submitted to exercise MPI (*n* = 228), NCPS were higher during stress than at rest (3.52 ± 2.03 vs. 2.78 ± 2.07, respectively; *p* < 0.01). This effect was not detected in the 58 patients submitted to dipyridamole-stress. The response to exercise selectively involved the spleen, since NCPS in heart were unchanged irrespective of the used stressor. This same response was dependent upon gender, indeed spleen NCPS during stress were significantly higher in the 75 women than in the 153 men (3.86 ± 1.8 vs. 3.23 ± 1.6, respectively, *p* < 0.01). Again, this variance was not reproduced by heart. Finally, spleen NCPS were lower in the 173 patients with myocardial reversible perfusion defects (summed difference score ≥3) than in the remaining 55, despite similar values of rate pressure product at tracer injection.

**Conclusion:** Thus, exercise interference on spleen perfusion can be detected during MPI. This effect is dependent upon gender and ischemia confirming the high sensitivity of this organ to SNS activation.

## Introduction

In patients with coronary artery disease (CAD), the activation of the sympathetic nervous system (SNS) contributes to trigger ischemic episodes during physical effort, since the sudden increase in arterial pressure, heart rate, and cardiac contractility augment myocardial oxygen consumption, possibly leading to an inadequate blood flow supply downstream from a severe coronary stenosis (L'Abbate et al., 1999).

The capability to inhibit these SNS effects justifies the wide clinical use of beta-blocking agents in anginal patients. On the other hand, the effectiveness of these drugs has been found, in most circumstances, to be less evident in women than men (Packer et al., 1996; Study Group and H. F., 1999; Wilmot et al., 2015; EUGenMed Cardiovascular Clinical Study Group et al., 2016; Mehta et al., 2016; Bots and Peters, 2017). This difference agrees with the divergent SNS modulation of systemic hemodynamics between women and men that has been observed both in humans (Hinojosa-Laborde et al., 1999; Weitz et al., 2001; Dart and Du, 2002; Hart et al., 2009; Momen et al., 2010; Briant and Charkoudian, 2016) and experimental models (Luksha et al., 2005). It thus raises the need for a more detailed comprehension of the “gender specificity” of sympathetic response to systemic signals.

Sympathetic activation has been most intensively studied by the evaluation of blood pressure, heart rate, and their variability during exercise (O'Toole, 1989; Gleim et al., 1991; Aubert and Seps, 2003; Ozdemir et al., 2003; Kappus et al., 2015; Yoo, 2020). Nevertheless, the SNS contribution to the “fight or flight” response (Cannon, 1929) is not limited to the regulation of heart function. Rather, it extends to encompass a series of variegate effects able to modify metabolic pattern and microvascular resistance distribution in a series of districts (Perko et al., 1998; Pan et al., 2001), such as skeletal muscles (Mortensen, 2014), kidneys (Schlader et al., 2019), gastrointestinal tract (Ter Steege, 2012), and the splanchnic district (Bakovic et al., 2013a,b; Mizuno, 2017).

Among these targets, a peculiar role is played by the spleen whose response to exercise reflects an α-mediated contraction of smooth muscle cells (SMC) of vasculature and capsule (Pinkus et al., 1986; Agostoni et al., 1999; Engan et al., 2014). This response favors a blood delivery into systemic circulation that has been extensively characterized (Stewart and McKenzie, 2002; Stewart et al., 2003). This response is scarcely counterbalanced by the parasympathetic activation due to the virtual local absence of cholinergic receptors (Ayers and Davies, 1972; Mignini and Streccioni, 2003; Shephard, 2003; Verlinden et al., 2019). Coupled with the metabolic response to activation β2-adrenoceptors, the α-mediated SMC contraction raises the oxygen consumption of the splenic parenchyma and thus should imply an increased flow rate.

So far, this hypothesis has not been tested in the clinical setting. Nevertheless, the spleen is almost invariably included, partially or in toto, in the field of view of stress and rest images of myocardial perfusion imaging (MPI). This inclusion allows the measurement of organ uptake of ^99m^Tc-labeled perfusion tracers and thus the semiquantitative estimation of spleen perfusion response to exercise. Thus, the present study aimed to evaluate whether exercise does modulate spleen uptake of ^99m^Tc-labeled Sestamibi, as an index of the splanchnic SNS drive and whether gender plays a significant role in this response.

## Materials and Methods

Stress and rest original scans of all 286 consecutive patients, submitted to MPI in the course of the year 2019, were collected and evaluated. All patients were in sinus rhythm. Subjects with atrial fibrillation or pacemaker were excluded from the analysis as to warrant a precise measurement of scan duration. Tracer was injected with the patient approaching the maximal workload or 2 min after the administration of standard dipyridamole dose (0.56 mg/Kg body weight over 4 min). Both stresses were performed under continuous electrocardiographic monitoring (EKG). A 12-derivations printout and arterial pressure were recorded each minute. In all cases, the following clinical parameters were recorded: rate pressure product (RPP) at baseline and at tracer injection, occurrence of chest pain or dyspnea as well as appearance of ST segment depression.

### Myocardial Perfusion Imaging

According to our standard procedure, all patients underwent a single-day stress-rest sequence with a gap of ≥2 h between the administrations of 180 and 500 MBq of ^99m^Tc-Sestamibi, respectively. Imaging was performed 30 min after radiotracer administration, with scan duration set at 35 and 25 s per view for stress and rest acquisition, respectively. In all cases, single photon computerized tomography (SPECT) was acquired using a dual-head SPECT gamma camera (Discovery NM630, GE Healthcare, Chicago, IL, USA) equipped with parallel hole low-energy high-resolution collimators.

The energy window (10%) was centered over the 140 keV ^99m^Tc photopeak. An automated body-contour orbit was used, with image acquisition every 3 degrees, through a 180 degrees arc starting from 45 degrees right anterior oblique to 45 degrees left posterior oblique view, with heads configured in L mode. No attenuation or motion correction was applied. According to the current guidelines (Hesse et al., 2005), SPECT scans were performed using both non-gated and gated protocols. Acquisition matrix was 64 × 64, while for EKG-gating cardiac cycle was divided into 12 frames. In all scans, at least the cranial half of spleen was included allowing the analysis of average tracer content in these organs.

### SPECT Scans Processing

All scans were analyzed by using a commercial software (Myovation Evolution, GE Healthcare, Chicago, IL, USA). To minimize the possible differences in reconstruction parameters, non-gated images were reconstructed using the filtered back-projection method with Butterworth filter set at 0.5 frequency and 8th order.

Radioactivity content was only measured in non-gated transaxial images. To this purpose, two volumes of interest (≥10 ml) were drawn to estimate average counts of spleen and left ventricular (LV) myocardium. Obtained values were thus divided for the injected dose, and the scan duration to obtain the normalized counting rate (normalized counts per second, or NCPS). No attenuation-correction was performed.

Myocardial perfusion imaging was semi-quantitatively evaluated using the commercial QPS/QGS software (Cedars QPS/QGS, Los Angeles, CA, USA). The left ventricle was divided into 20 segments to automatically calculate the summed stress score (SSS), summed rest score (SRS), and summed difference score (SDS) for each patient. Ischemia was defined by a SDS value ≥3.

The EKG-gated images were reconstructed using the ordered subset expectation maximization method and applying the correction for the line-spread function. LV end-diastolic volume (EDV) and ejection fraction (EF) values were thus estimated using the dedicated routine of QGS software.

### Statistical Analysis

Continuous data are expressed as mean ± SD, prevalence is described as *n* and percentage. For continuous variables, differences between the groups were tested using the Student's *t*-test for unpaired or paired data as appropriate. Difference in prevalence was tested by chi-square test. Univariate regression was performed to assess the value of clinical and imaging data in predicting spleen uptake after exercise-induced stress. Thereafter, all variables were tentatively included in a multivariate regression model by means of a step-back (backward) procedure, based on the likelihood ratio test and thus using a *p* < 0.15 for the inclusion of each variable. Data analysis was performed by using the IBM Statistical Package SPSS version 26 (IBM Corp., Armonk, NY, USA). Statistical significance was set at *p* < 0.05.

## Results

### Exercise vs. Dipyridamole-Induced Stress

Among the entire population, 185 (65%) patients were men and 101 (35%) women. Exercise was accomplished in 228 subjects: 153 (67%) of them were men and 75 (33%) women. Fifty-eight patients were submitted to pharmacologic vasodilation: 32 (55%) men and 26 (45%) women.

Table 1 displays the demographic and clinical features of the study population. Prevalence of pharmacologic stress was similar in women and men. Prevalence of patients with documented CAD and submitted to MPI to characterize site and severity of myocardial ischemia was equally distributed between the two stressors. By contrast, physical exercise was performed in significantly younger patients and caused a more evident SNS activation, as testified by the higher increase in RPP at tracer injection. Similarly, it was associated with a higher prevalence of active beta-blocking treatment and a higher incidence of EKG abnormalities, with respect to dipyridamole. By contrast, the occurrence of symptoms was equally distributed in the two groups.

**Table 1:** Demographic and clinical characteristics of patients grouped by the myocardial perfusion imaging (MPI) stressor used.

	**Overall** **(***n*** = 286)**		**Exercise** **(***n*** = 228)**		**Dipyridamole** **(***n*** = 58)**		**Sig**.
Age	67.34 ± 11.90		66.33 ± 11.34		71.33 ± 13.26		*p* < 0.01
Female	101	35%	75	33%	26	45%	*p =* ns
Documented CAD	112	39%	87	38%	25	43%	*p =* ns
Beta-blockade	117	41%	108	47%	9	16%	*p <* 0.0001
Workload (Watt)	not reported		94.01 ± 31.67		not reported		
Max RPP (mmHg beats min^−1^)	not reported		21,557 ± 5,166		11,431 ± 2,582		*p <* 0.01
Rest RPP (mmHg beat min^−1^)	9,051 ± 1,712		9,139 ± 1,831		8,702 ± 1,561		*p =* ns
Stress-induced symptoms	23	8%	16	7%	7	12%	*p =* ns
Transient EKG alterations	74	26%	74	33%	0	0%	*p* < 0.0001
Post-stress LV EF (%)	60.26 ± 12.02		60.61 ± 11.76		58.82 ± 13.03		*p =* ns
Rest LV EF (%)	61.20 ± 12.15		61.56 ± 12.10		59.73 ± 12.31		*p =* ns
Post-stress LV EDV (mL)	97.94 ± 41.48		97.29 ± 41.64		100.57 ± 41.11		*p =* ns
Rest LV EDV (mL)	93.45 ± 36.99		93.01 ± 36.64		95.25 ± 38.64		*p =* ns
SSS	8.11 ±7.51		7.94 ± 7.35		8.76 ± 8.16		*p =* ns
SRS	2.90 ± 5.34		2.82 ± 5.36		3.21 ± 5.28		*p =* ns
SDS	5.21 ± 3.89		5.12 ± 3.82		5.55 ± 4.17		*p =* ns
SDS ≥3	215	75%	173	76%	42	72%	*p =* ns

Contractile function was not different in the two groups, since post-stress and baseline LV EF were similar in subjects submitted to exercise or dipyridamole. Likewise, prevalence of positive MPI as well as numerical descriptions of MPI results (SSS, SRS, and SDS) were independent of the used stressors.

### Spleen Uptake and Exercise

**Figure 1:**
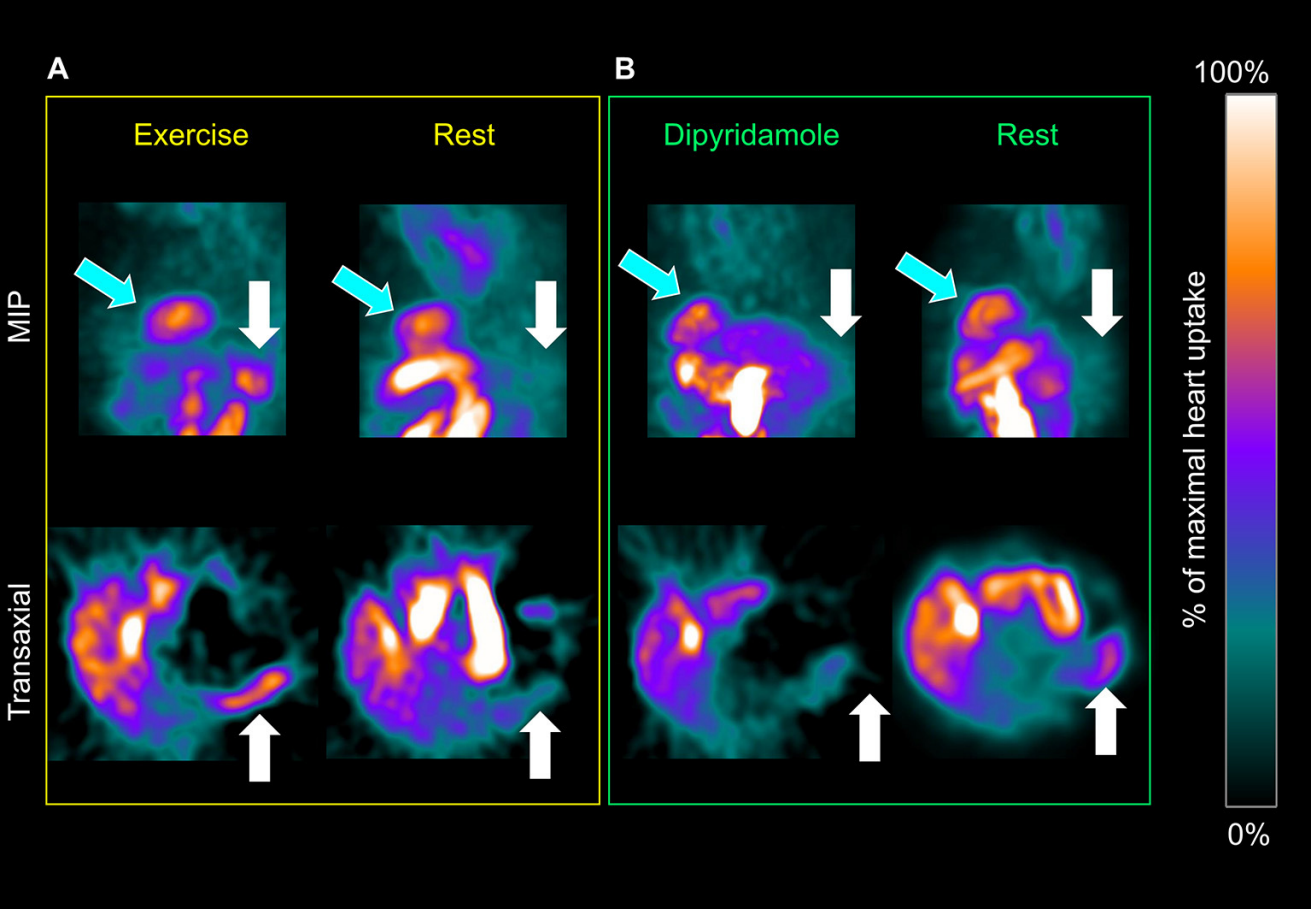
Maximal intensity projection (top row) and transaxial planes (bottom) of a patient submitted to exercise and rest **(A)** or dipyridamole and rest **(B)** myocardial perfusion imaging (MPI). Spleen tracer uptake (white arrows) is markedly increased by exercise and scarcely influenced by pharmacological vasodilation. This effect is not apparent when the heart (light blue arrows) is considered.

**Figure 2:**
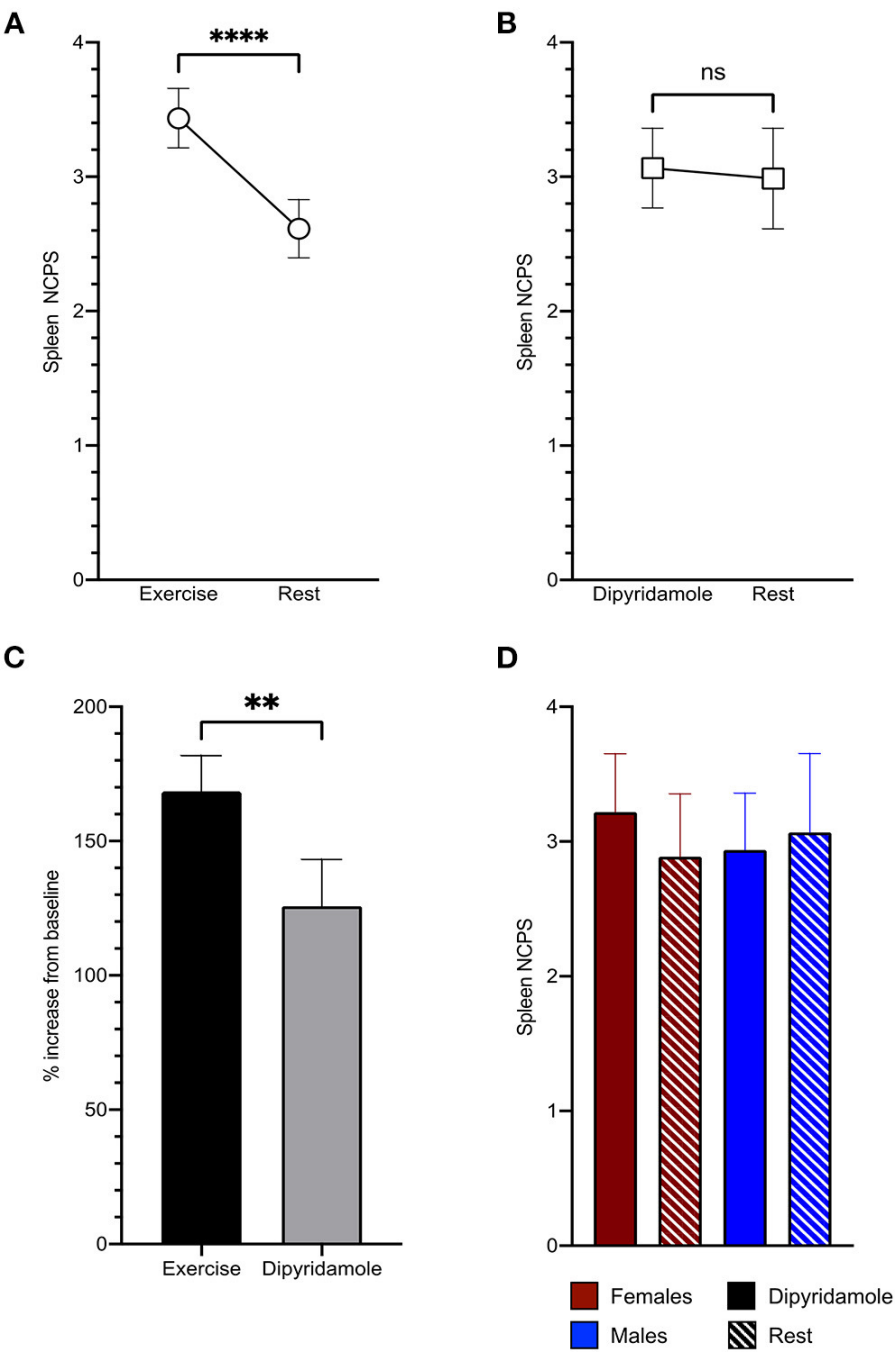
Spleen tracer uptake is significantly increased by exercise **(A)** and scarcely influenced by dipyridamole **(B)**. The percentage of increase of spleen normalized counts per seconds (NCPS) between stress and rest is significantly higher in patients submitted to exercise than to pharmacological vasodilation **(C)**. In the dipyridamole group **(D)**, both women (red) and men (blue) show no significant differences of spleen tracer uptake between stress (solid columns) and rest (striped columns). **p < 0.01, ****p < 0.0001, and ns = not significant.

Spleen ^99m^Tc-labeled Sestamibi uptake was clearly visible in all studied patients both in stress and rest images (Figures 1A,B). In the 228 patients submitted to exercise, physical effort increased spleen tracer retention with respect to rest (3.44 ± 1.7 vs. 2.61 ± 1.66 NCPS, respectively; *p* < 0.0001) (Figures 1A, 2A). This difference was not reproduced in subjects submitted to pharmacologic vasodilation, in whom spleen radioactivity was superimposable in stress and rest images (3.06 ± 1.13 vs. 2.98 ± 1.42 NCPS, respectively; *p* = ns) (Figures 1B, 2B). Therefore, the increase of spleen tracer uptake was significantly higher in the exercise group than in the dipyridamole one (169 ± 101% vs. 126 ± 66%, respectively; *p* < 0.01) (Figure 2C).

As shown in Figure 2D, the absent response of spleen tracer uptake to dipyridamole was shared by both men and women. We thus concluded that spleen ^99m^Tc-labeled Sestamibi retention was selectively increased by physical effort and focused our attention on the determinants of organ radioactivity content in the 228 patients submitted to exercise MPI.

### Ischemia as a Determinant of Spleen Uptake During Exercise

Reversible perfusion defects, defined by an SDS ≥ 3, occurred in 173/228 (76%) patients submitted to exercise MPI. As reported in Table 2, these subjects showed lower LV EF values and larger LV EDV both at rest and after exercise. Nevertheless, the occurrence of ischemia was not associated with lower values of maximal workload, maximal RPP, or ratio between stress and rest RPP indicating a similar degree of SNS activation with respect to non-ischemic patients.

**Table 2:** Demographic and clinical characteristics of patients submitted to exercise MPI grouped by presence or absence of scintigraphic evidence of myocardial ischemia (summed difference score [SDS] ≥ 3).

	**SDS ≥ 3**		**SDS < 3**		**Sig**.
	**(***n*** = 173)**		**(***n*** = 55)**		
Age	66.34 ± 11.31		66.29 ± 11.54		*p =* ns
Female	43	25%	32	58%	*p* < 0.0001
Documented CAD	75	43%	12	21%	*p* < 0.01
Beta-blockade	86	50%	22	40%	*p =* ns
Workload (Watt)	96.30 ± 33.28		86.82 ± 24.93		*p =* ns
Max RPP (mmHg beats min^−1^)	21,593 ± 5,176		21,443 ± 5,183		*p =* ns
Rest RPP (mmHg beat min^−1^)	9,154 ± 1,921		9,091 ± 1,766		*p =* ns
Stress-induced symptoms	15	9%	1	2%	*p =* ns
Transient EKG alterations	58	34%	16	29%	*p =* ns
Post-stress LV EF (%)	59.06 ± 12.12		65.57 ± 8.97		*p* < 0.0001
Rest LV EF (%)	60.36 ± 12.33		65.31 ± 10.60		*p* < 0.01
Post-stress LV EDV (mL)	103.01 ± 45.41		78.96 ± 15.91		*p* < 0.0001
Rest LV EDV (mL)	97.74 ± 40.19		78.20 ± 14.41		<0.001
SSS	9.62 ± 7.42		2.65 ± 3.74		*p* < 0.0001
SRS	3.18 ± 5.76		1.67 ± 3.69		*p =* ns
SDS	6.44 ± 3.43		0.98 ± 0.95		*p* < 0.0001

**Figure 3:**
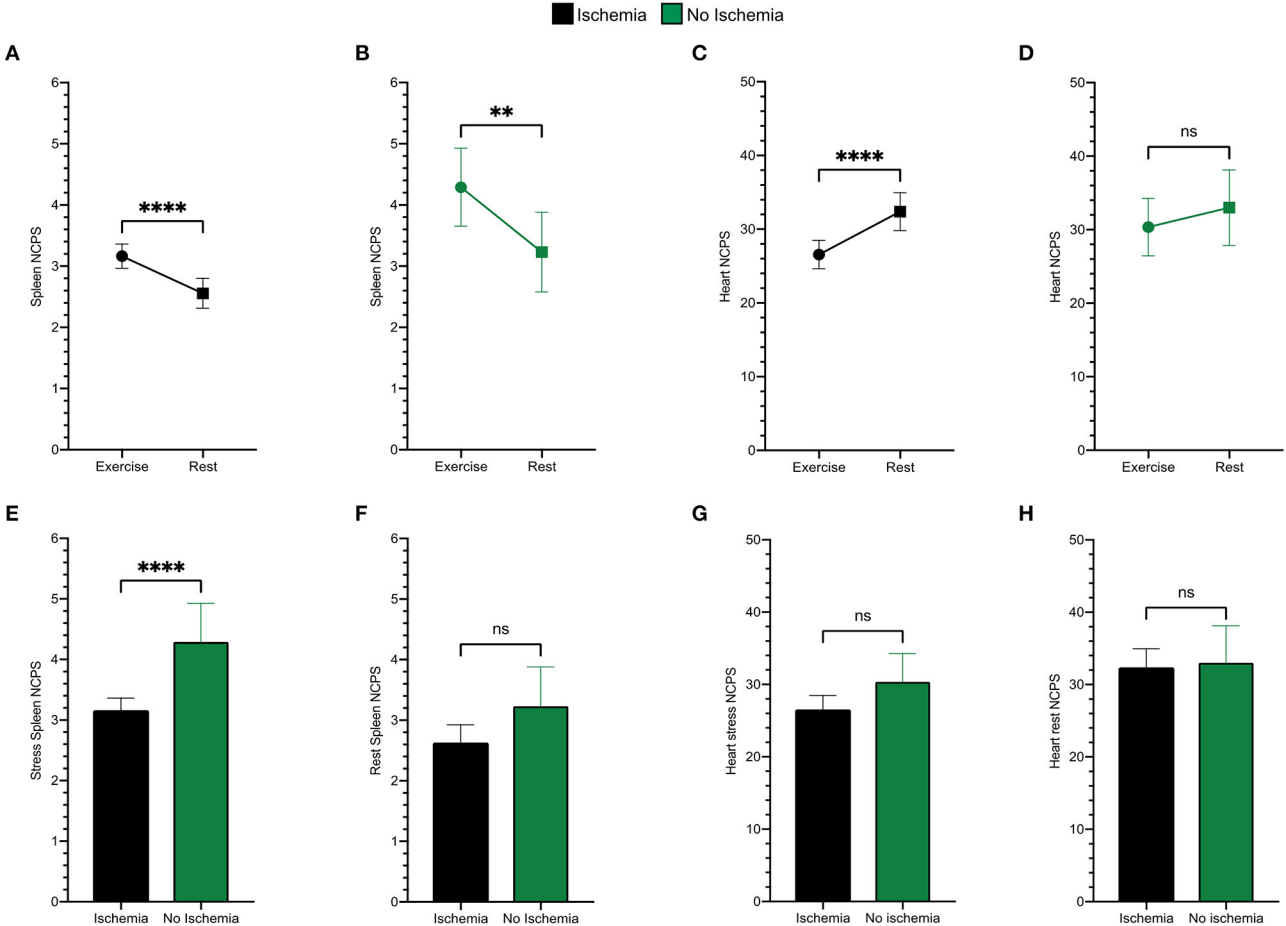
Spleen tracer uptake is markedly increased by exercise both in ischemic (black) **(A)** and in non-ischemic (green) **(B)** patients. An opposite trend is evident for the variation of the heart NCPS in patients presenting ischemia **(C)**, while differences between stress and rest cardiac uptake are not significant in the non-ischemic ones **(D)**. Patients presenting ischemia show significantly lower spleen NCPS after exercise (**E**), while they present superimposable values at rest **(F)**. The cardiac tracer uptake results similar in both groups after exercise **(G)** and at rest **(H)**. ^**^p < 0.01, ^****^p < 0.0001, and ns = not significant.

As shown in Figure 3, stress spleen tracer concentration was significantly increased by exercise in both ischemic and non-ischemic patients (Figures 3A,B). By contrast, exercise decreased myocardial ^99m^Tc-labeled Sestamibi uptake in ischemic patients (Figure 3C), while this reduction was only mild and not significant in non-ischemic ones (Figure 3D).

When analysis focused on stress images, the spleen radioactivity concentration was lower in patients with an SDS ≥ 3 than in the remaining ones (Figure 3E) despite a similar tracer retention at rest images (Figure 3F). By contrast, this difference was less evident in the myocardium which average NCPS were virtually independent of the susceptibility to ischemia at both stress and rest MPI (Figures 3G,H).

### Gender as a Determinant of Spleen Uptake Response to Exercise

As detailed in Table 3, exercise MPI included 75 women and 153 men, allowing the evaluation of gender interference on spleen response to exercise. Age was remarkably similar in the two genders. Among women, menopause was largely prevalent, as it characterized 62/75 (83%) women aging >55 years. Overall, women showed a lower prevalence of subjects with pre-test documentation of CAD, higher LV EF, and lower EDV values with respect to men. Likewise, female gender was also associated with lower values of SSS, SDS, and SRS.

**Table 3:** Demographic and clinical characteristics of patients submitted to exercise MPI grouped by gender.

	**Female**		**Male**		**Sig**.
	**(***n*** = 75)**		**(***n*** = 153)**		
Age	66.14± 10.32		66.42 ± 11.84		*p =* ns
Documented CAD	13	17%	74	48%	*p* < 0.0001
Beta-blockade	31	41%	77	50%	*p =* ns
Workload (Watt)	77.33 ± 21.42		102.19 ± 32.72		*p* < 0.0001
Max RPP (mmHg beats min^−1^)	21,943 ± 5,823		21,367 ± 4,821		*p =* ns
Rest RPP (mmHg beat min^−1^)	9,180 ± 1,787		9,118 ± 1,931		*p =* ns
Stress-induced symptoms	4	5%	12	8%	*p =* ns
Transient EKG alterations	28	37%	46	30%	*p =* ns
Post-stress LV EF (%)	65.33 ± 10.47		58.28 ± 11.70		*p* < 0.0001
Rest LV EF (%)	66.01 ± 11.07		59.36 ± 12.01		*p* < 0.0001
Post-stress LV EDV (mL)	77.92 ± 22.62		106.85 ± 45.45		*p* < 0.0001
Rest LV EDV (mL)	76.33 ± 18.52		101.24 ± 40.43		*p* < 0.0001
SSS	4.91 ± 5.35		9.43 ± 7.74		*p* < 0.0001
SRS	1.00 ± 2.57		3.71 ± 6.11		*p* < 0.0001
SDS	3.91 ± 3.53		5.72 ± 3.83		*p* < 0.001
SDS ≥ 3	43	57%	130	85%	*p* < 0.0001

**Figure 4:**
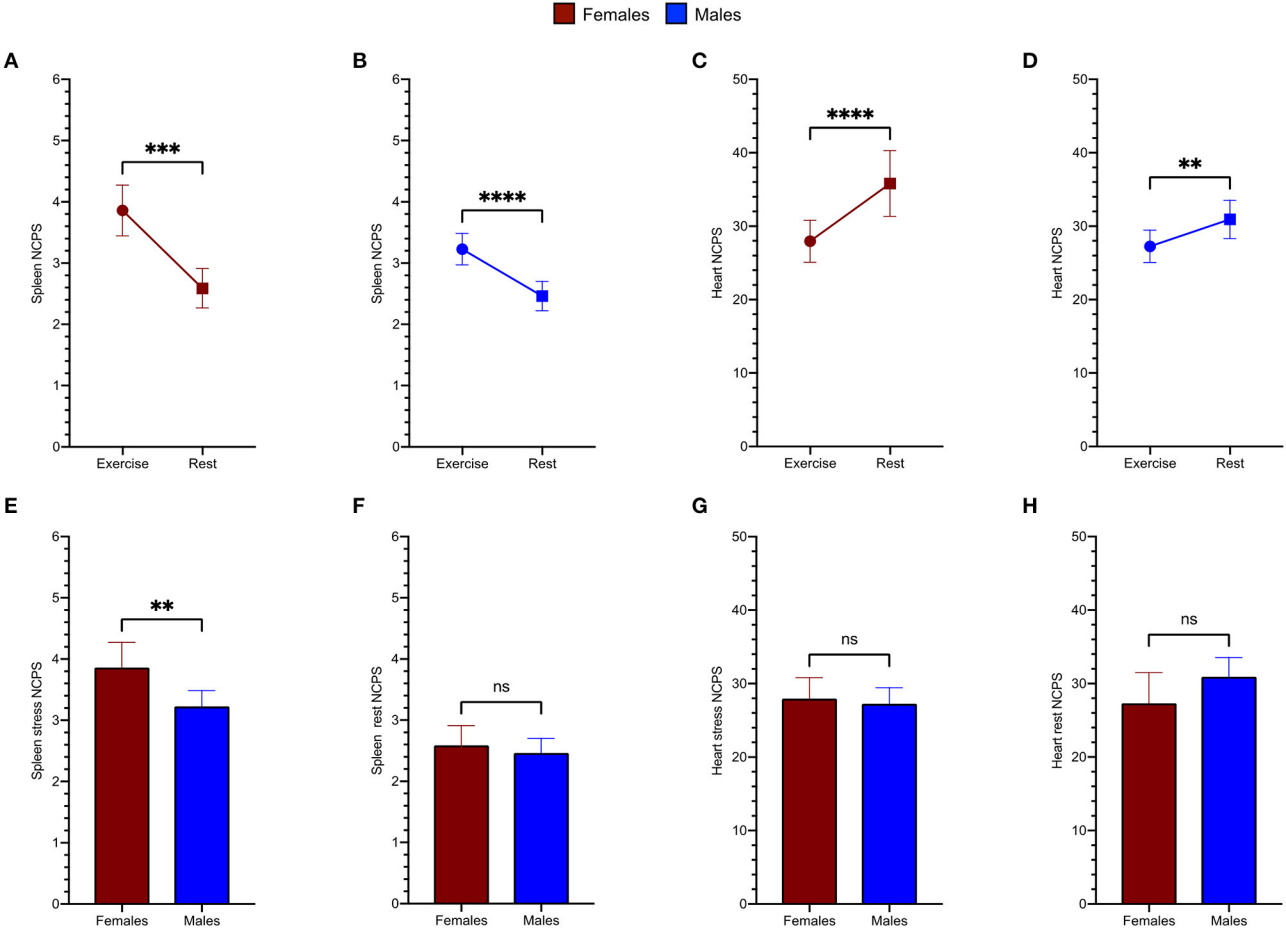
Spleen tracer uptake is markedly increased by exercise both in women (red) **(A)** and in men (blue) **(B)**. An opposite trend is evident for the variation of the heart NCPS in the female group **(C)** as well as in male patients (**D**). Women show remarkably higher spleen NCPS after exercise **(E)**, while they present superimposable values at rest **(F)**. The cardiac tracer uptake results similar in both groups after exercise **(G)** and at rest **(H)**.

As shown in Figure 4, spleen radioactivity content significantly increased both in men and women from baseline to exercise (Figures 4A,B). On the other hand, the decrease of myocardial tracer uptake during exercise was gender independent (Figures 4C,D). However, when stress images were considered, spleen radioactivity concentration was higher in women with respect to men, Figure 4E despite similar counting rates at baseline scans (Figure 4F). Again, this difference selectively involved the spleen, since the heart counting rate (Figures 4C,D) were similar in both women and men regardless of the studied condition (Figures 4G,H).

To verify the role of gender in spleen response to exercise, we performed a univariate analysis including as possible determinants of spleen trace uptake the following variable: age, gender, SDS, active betablocker treatment, and LV EF values at rest or after stress. As displayed in Table 4A, SDS and gender resulted as the only reliable predictors of stress spleen radioactivity concentration. The multivariate analysis confirmed the independent prediction of spleen response to stress provided by gender and ischemia (Table 4B).

**Table 4:** Univariate **(A)** and multivariate **(B)** general linear model with spleen NCPS after stress set as dependent variable between patients submitted to exercise MPI.

**A**	**Unstandardized coefficients**		**Standardized coefficients**			**95% Confidence interval for B**	
	**B**	**Std. error**	**Beta**	**t**	**Sig**.	**Lower bound**	**Upper bound**
Age	0.004	0.010	0.028	0.423	0.673	−0.015	0.024
Female gender	0.632	0.236	0.175	2.678	0.008	0.167	1.098
Beta-blockade	−0.048	0.226	−0.014	−0.214	0.831	−0.493	0.396
Post-stress LV EF	0.025	0.027	0.171	0.924	0.356	−0.028	0.077
Rest LV EF	−0.006	0.026	−0.044	−0.239	0.811	−0.057	0.045
SDS	−0.092	0.029	−0.208	−3.192	0.002	−0.149	−0.035
**B**	**Unstandardized coefficients**		**Standardized coefficients**			**95% Confidence interval for B**	
	**B**	**Std. error**	**Beta**	**t**	**Sig**.	**Lower bound**	**Upper bound**
Female gender	0.490	0.239	0.136	2.049	0.042	0.019	0.961
SDS	−0.079	0.029	−0.177	−2.676	0.008	−0.137	−0.021

## Discussion

The main finding of the present study is the evidence of a significant increase in spleen ^99m^Tc-labeled Sestamibi uptake during exercise. Coupled with the absent effect of dipyridamole stress, this observation indicates that spleen radioactivity concentration is dependent on physical effort. The occurrence of ischemia during exercise partially prevented this response, and eventually resulted in a lower tracer retention selectively involving the spleen and not the myocardium. Altogether, these findings suggest that splenic uptake of a perfusion tracer reflects the SNS activation by exercise and its response to ischemia.

This response was evident in both genders. However, differently from myocardial behavior, spleen increase in tracer retention was higher in women than in men despite a similar radioactivity concentration in rest imaging. This finding suggests a selective interference of genders on sympathetic fibers innervating the splanchnic district, with a less appreciable impact on the sympathetic innervation of the LV myocardium and, in general, the heart.

### ^99m^Tc-Sestamibi Uptake and Tissue Perfusion in the Spleen

Exercise effect on radioactivity content of spleen and heart was estimated by normalizing tissue counts for injected dose and scan duration. ^99m^Tc-Sestamibi is a lipophilic cation that binds to the mitochondrial membranes of reached cells (Piwnica-Worms and Kronauge, 1990). According to the Sapirstein fractionation principle, bound tracer is thus a function of the number of labeled molecules delivered to each tissue, i.e., the ratio between local blood flow and cardiac output (Sapirstein, 1956). Thus, the increased tracer content can reasonably be considered an index of an increased spleen perfusion in response to the physical effort. By contrast, the simultaneous decrease in heart tracer content is coherent with the notion that, due to its high rate at rest, coronary blood flow response to physical effort was comparable with the corresponding increase in cardiac output. This interpretation is further corroborated by the virtual absent response of spleen ^99m^Tc-Sestamibi uptake to dipyridamole that agreed with the local flow reduction documented by PET and ^82^Rb (Keramida and Gregg, 2020) and by MRI during adenosine stress (Patriki et al., 2021).

Altogether, these observations configure spleen ^99m^Tc-Sestamibi uptake as an index of tissue perfusion, whose response to exercise would be independent (or even inversely correlated) with the organ blood content. This observation extends to humans as the previous evidence of an almost six-fold increase in splenic blood flow during exercise obtained using the microsphere method in rats (Maeda et al., 2002). On the other hand, it extends our comprehension of perfusion response to physical efforts in the splanchnic district indicating that the reduction in global flow documented by the evaluation of indocyanine concentration in hepatic veins (Kenney, 1995; Ho et al., 1997) most likely reflects an increase in microvascular resistance of organs other than the spleen.

### Spleen Perfusion and SNS Function

Although the underlying mechanisms were not investigated, the increased splenic flow during exercise is coherent with the metabolic response to the sympathetic firing associated with physical effort. Indeed, a wide literature (Stewart and McKenzie, 2002; Engan et al., 2014; Shephard, 2016) documented that the blood reservoir function of this organ is modulated by the SNS whose response to physical effort activates both α- and β-adrenergic receptors (Ignarro, 1968) causing an SMC contraction as a function of exercise intensity and duration (Stewart et al., 2003). The consequent metabolic activation would thus inevitably raise the oxygen demand and thus the flow rate through the splenic parenchyma.

According to these considerations, spleen perfusion, and thus ^99m^Tc-Sestamibi uptake, might represent an index of local firing by sympathetic nerves. At least two observations in the present study corroborate this hypothesis. On one side, the scarce response of spleen tracer concentration to dipyridamole closely agrees with the finding of an only modest increase or even a downregulation of SNS activity (Hom, 1981) after its administration. On the other side, the reversible myocardial perfusion defects were found to represent a main determinant of spleen radioactivity content at stress images regardless of the exercise intensity and maximal RPP. Again, this link closely agrees with the well documented notion that myocardial ischemia can trigger an intense sympathetic response with potentially life-threatening consequences (Figueras, 1981; Longhurst et al., 2001) with the spleen playing a relevant role in this complex series of reflexes (Heusch, 2019).

Accordingly, the present observations support the notion that spleen perfusion largely regulated by sympathetic drive. On the other hand, they indicate that SNS function might differently modulate the cardiac and the splanchnic districts with reciprocal interferences that still need to be defined despite their potential impact that has been described both in experimental models and patients (Rein and Dohrn, 1951; Tarasiuk and Sasson, 2000).

### Gender Differences in Response to Exercise

Sympathetic nervous system plays a pivotal role in the cardiovascular adaptation to the sudden increase in metabolic demand caused by exercise. The noradrenergic signaling indeed increases heart rate and myocardial contractility but also extends to redistribute blood flow away from “passive” visceral organs toward “active” skeletal and cardiac muscles. This integrated response is modulated by a variety of signals that can differently affect perfusion rate in the various tissues depending on coexistent morbidities, lifestyle, gender, and age. According to this consideration, literature about gender differences in vascular response to exercise mostly focused on flow response to the active muscle of both upper and lower limbs. These evaluations showed that aging is associated with both a progressive increase in SNS activity (Sundlöf, 1978; Narkiewicz et al., 2005; Smith et al., 2007; Best et al., 2014; Hearon and Jr, 2016; Barnes, 2021) and a progressive impairment in the capability of metabolic signals to prevent the α-mediated vasoconstriction in young subjects (Dinenno and Dietz, 2002; Dinenno and Masuki, 2005; Dinenno, 2006; Hearon and Jr, 2016). Though present in both genders, both processes are enhanced in post-menopausal women (Kruse et al., 2018) with respect to aging men and are particularly evident during exercise (Maeda et al., 2002; Moreau et al., 2003; Moreau, 2017).

Our clinical study did not include the legs in the acquired field of view and thus did not permit to evaluate the blood flow response in exercising skeletal muscles. Nevertheless, the selective increase in spleen tracer uptake in women extends these previous studies, by documenting that gender interference on flow response to exercise extends to this splanchnic district. Although opposite in sign, this finding might reflect the same sex-interference on SNS activation. Indeed, the α-mediated contraction of SMC extends to involve the organ capsule and represents, in this parenchyma, the main determinant of tissue metabolic demand. This physiologic feature combines with the extremely low perfusion rate under baseline conditions and the pronounced flow response to the contraction elicited by the sympathetic firing during exercise (Maeda et al., 2002). Thus, whether confirmed by studies directly focused on the response of splanchnic SNS to exercise in women and in men, this piece of evidence might indicate a further—so far not explored—gender-related difference in SNS function and its regulation.

Obviously, the observational nature of our study does not permit to verify the nature of the link between SNS activity and spleen flow response to exercise. Nevertheless, the largely divergent organ tracer uptake in the two genders suggests the need for a more detailed comprehension of the mechanisms underlying this finding as a possible new window to understand the different prognostic impact of cardiovascular syndromes in women and men.

### Limitations

The main limitation of the present study is intrinsic to the relatively rough nature of the adopted methodology. Indeed, the physical features of SPECT do not allow an estimation of arterial input function and, thus, do not permit to convert the tissue radioactivity concentration in indexes related to the local blood flow. Similarly, counting rate of single photon emitting radionuclides is dependent upon tissue attenuation and, more importantly, on the distance between the radioactive source and the detector. Both these factors were undoubtedly lower and might have resulted in apparently higher spleen tracer concentrations in women with respect to men.

Nevertheless, the interference by these technical confounders must be comparable in rest and stress images. Likewise, it should hamper to a similar extent the evaluation of both heart and spleen. In the present data, gender and occurrence of myocardial ischemia were associated with different spleen radioactivity content selectively in stress images. By contrast, organ counting rates were similar under resting condition in both men and women as well as in ischemic or non-ischemic patients. Similarly, the response of spleen radioactivity was completely independent of the corresponding pattern observed in the myocardium at the same scan acquisition.

A similar concern relies on our clinical protocol, whose 1-day nature prevents an accurate estimation of resting spleen tracer uptake. Indeed, the possible contamination by the residual stress radioactive content prevents to verify the influence of clinical variables on spleen ^99m^Tc-Sestamibi uptake under resting conditions. However, stress-related differences disappeared in all groups when rest images were evaluated, suggesting a scarce relevance of this potential pitfall.

Accordingly, the quoted limitations scarcely apply to the population analysis described in the present study. Nevertheless, they indicate the need of a careful work to evaluate the response of spleen blood flow to exercise as a variable of possible clinical interest.

### Clinical Implications and Conclusion

The relevance of the present findings relies on the direct interaction between spleen and heart, that has been found since the late 1940s (Rein, 1949) with the evidence of an improved myocardial contractility in the presence of hypoxia, after stimulation of splenic nerves. This concept has been subsequently corroborated by the evidence of an increased long-term death rate after myocardial infarction, in World War II veterans submitted to splenectomy (Robinette, 1977). On the other hand, in patients with acute coronary syndromes, spleen metabolic activation has been found to predict a higher cardiovascular risk (Emami et al., 2015). Although the underlying mechanisms are far beyond the scope of our work, this interference seems to involve the increased splenic β2-adrenoceptors-mediated production of interleukin 10 (Tian et al., 2018) in the early phases of myocardial infarction, as well as the high contribution of the spleen in the overall activity of angiotensin-converting enzyme (Swirski et al., 2009).

In the present study, splenic response to exercise was more pronounced in women than in men. This difference was not paralleled by the response of commonly evaluated indexes of cardiac sympathetic response (i.e., heart rate and arterial pressure) that were gender independent. This observation suggests that SNS response to exercise might be more variegate than commonly assumed, with differential activations of sympathetic neurons regulating the splanchnic district with respect to the heart function.

In conclusion, the present proof of concept study documented a marked increase in spleen ^99m^Tc-Sestamibi uptake during exercise. This response was not shared by patients submitted to dipyridamole stress. It is coherent with previous experimental evidence about an increased flow rate during physical effort and most likely reflects the consequence of an empowered adrenergic and noradrenergic signaling by the sympathetic fibers.

Differently from the myocardium, spleen response to exercise was at least partially dependent upon gender and occurrence of ischemia, possibly indicating a divergent reaction to exercise by cardiac and splanchnic SNS. Should these data be confirmed, the evaluation of SNS regulation of perfusion and metabolic function of the spleen might represent a new window to study the reasons underlying the higher risk of women with coronary artery disease (Dart and Du, 2002; Momen et al., 2010; Wilmot et al., 2015; EUGenMed Cardiovascular Clinical Study Group et al., 2016; Mehta et al., 2016; Bots and Peters, 2017), as well the divergent benefit provided by the current therapeutic approach to LV dysfunction.

## Data Availability Statement

The datasets generated for this study are available on request to the corresponding author.

## Ethics Statement

Ethical review and approval was not required for the study on human participants in accordance with the local legislation and institutional requirements. The patients/participants provided their written informed consent to participate in this study.

## Author Contributions

FL, FD, GS, and CM: conceptualization and original draft preparation. SR, MP, MD, AM, SC, SM, and CD: images acquisition. FL and FD: data collection and analysis. All authors contributed to writing and editing and approved the definitive version of the manuscript.

## Conflict of Interest

The authors declare that the research was conducted in the absence of any commercial or financial relationships that could be construed as a potential conflict of interest.

## Publisher's Note

All claims expressed in this article are solely those of the authors and do not necessarily represent those of their affiliated organizations, or those of the publisher, the editors and the reviewers. Any product that may be evaluated in this article, or claim that may be made by its manufacturer, is not guaranteed or endorsed by the publisher.
